# Personalized sleep and nutritional strategies to combat adverse effects of night shift work: a controlled intervention protocol

**DOI:** 10.1186/s12889-024-20022-w

**Published:** 2024-09-19

**Authors:** Maaike van der Rhee, Johanneke E. Oosterman, Suzan Wopereis, Gijsbertus T. J. van der Horst, Inês Chaves, Martijn E. T. Dollé, Alex Burdorf, Linda W. M. van Kerkhof, Heidi M. Lammers-Van der Holst

**Affiliations:** 1 Department of Public Health, Erasmus MC, Rotterdam, 3000 CA The Netherlands; 2 Research Group Microbiology & Systems Biology, TNO, Organization for Applied Scientific Research, Leiden, 2333 BE The Netherlands; 3 Department Molecular Genetics, Erasmus MC, Rotterdam, 3000 CA The Netherlands; 4 Centre for Health Protection, National Institute for Public Health and the Environment (RIVM), Bilthoven, 3721 MA The Netherlands

**Keywords:** Shift work, Night shift, Circadian disruption, Sleep, Glucose homeostasis, Precision nutrition, Real-life intervention, Metabolic health, Occupational health

## Abstract

**Background:**

Working during the night interferes with the timing of normal daily activities and is associated with an increased risk of chronic diseases. Under controlled experimental conditions, interventions focusing on sleep and nutrition can mitigate the short-term adverse effects of shift work. However, it is unclear how these results translate to real-life, how they can be targeted to individual conditions, and how they relate to long-term health. Therefore, the current study aims to implement a personalized sleep and nutritional intervention among night shift workers in the field.

**Methods:**

A non-blinded controlled intervention study is used, consisting of a run-in period, an intervention of 3 months, post-intervention measurements, and a follow-up after 12 months. Three study arms are included: sleep intervention, nutritional intervention, and control group (*n* = 25 each). Participants are healthy 18–60-year male night shift workers, with at least one year of experience in night shift work. Information from the run-in period will be used to personalize the interventions. The main outcomes are sleep measurements and continuous interstitial glucose levels. Furthermore, general health biomarkers and parameters will be determined to further evaluate effects on long-term health.

**Discussion:**

This study aims to mitigate negative health consequences associated with night shift work by introducing two personalized preventive interventions. If proven effective, the personalized interventions may serve as practical solutions that can have a meaningful impact on the sustainable health and employability of night shift workers. This study will thereby contribute to the current need for high–quality data on preventative strategies for night shift work in a real-life context.

**Trial registration:**

This trial has been registered under ClinicalTrials.gov Identifier NCT06147089. Registered 27 November 2023.

## Introduction

Working during the night is associated with detrimental health effects such as increased risk of cardiovascular diseases, type 2 diabetes, respiratory infections, and cancer [1, 2]. Surveys estimate that approximately 19% of the workers in the European Union (EU) work during the night at least once per month [3], placing a considerable segment of the workforce at increased disease risk.

A widely supported hypothesis is that the misalignment between the endogenous circadian (i.e., near-24-h) rhythms and the timing of the sleep–wake cycle underlies the negative health effects associated with night shift work [4]. For example, the circadian system increases alertness during the daytime and decreases wakefulness during the nighttime [5]. This process is augmented by a homeostatic pressure for sleep, which builds across waking hours [6]. Normally, these two processes function in synchrony with the environmental light/dark cycle to maintain alertness while at work and sleep during the night. In contrast, night shift workers must work and sleep at times that conflict with their circadian rhythms.

Due to this altered sleep–wake cycle, eating, and activity, night shift work can lead to changes in physiology. For example shorter, less consolidated, and highly disrupted sleep has been observed in night shift workers during the day, while increased sleepiness and performance decrements are observed during night shifts [7]. Similarly, eating out of synchrony with the endogenous biological clock can lead to metabolic health problems, including alterations in glucose homeostasis such as tissue-specific insulin resistance [8, 9]. Furthermore, higher postprandial glucose concentrations may occur when eating during the night, in comparison to eating during the day [10].

As diet and sleep behavior are modifiable, it is important to understand how these factors might mediate the relationship between night shift work and health and how they can be implemented as countermeasures. Recent research shows promising results, as illustrated by the beneficial effect on cognitive performance and sleep duration around night shifts of power naps [11, 12] or limiting prior wakefulness (i.e. cumulative duration of wakefulness) [13]. Multiple sleep episodes can be as recuperative as a consolidated sleep episode [14–16]. As sleep insufficiency is the most widespread problem affecting night and rotating shift workers [17, 18], these strategies may be one way of minimizing overall daily sleep loss and associated declines in cardiovascular and metabolic health [19, 20]. Behavioral interventions focused on timing of nutrition mitigated the adverse effects in glucose regulation induced by a disrupted sleep–wake schedule [21, 22]. Furthermore, consuming a small meal during the night shift attenuates the increase in sleepiness and the decrease in vigor across the night shift, relative to consuming a larger meal or not eating during the night [23].

However, the limitation of these studies is that they focused only on short-term health effects such as fatigue and sleep [24], while evidence-based measures to minimize long-term health risks are currently lacking [25]. Furthermore, it is unclear how results obtained under controlled experimental conditions can be generalized to predict health effects in night shift workers in real-life [26, 27].

The adoption of a personalized approach may be a useful strategy to maximize health benefits in the field. Personalized interventions use individual-specific information to promote beneficial behavior change [28]. The personalized design results in greater dietary improvement compared to generic advice [29]. Furthermore, often a high degree of inter-individual metabolic responses to food intake is observed, as well as inter-individual differences in sleep responses to night shift work [30–32]. Accordingly, tailoring advice (based on objective data, as well as social context) may increase suitability of an intervention for each individual.

In summary, there is currently limited evidence on effective interventions in a real-life context to minimize short- as well as long-term health consequences of night shift work. As shift work is prevalent and difficult to limit in many job types, research into preventative interventions for night shift workers is an important avenue of investigation. Therefore, the current protocol describes a sleep or nutritional personalized intervention for night shift workers in a real-life. Our hypotheses are two-fold: i) the personalized sleep intervention can improve sleep duration and sleep quality in the field, particularly after night shifts, and ii) the personalized nutritional intervention can improve glucose metabolism in night shift workers. We expect both interventions to increase alertness, while decreasing fatigue during night shifts, in real life. If proven effective, the personalized interventions may serve as practical solutions that can have a meaningful impact on both short- and long-term health of night shift workers.

## Methods

### Study design

A non-blinded controlled intervention study will be used to investigate the effect of a personalized sleep or nutritional intervention. The study will include a run-in period with baseline levels, a 3-month intervention with measurements at the start of the intervention, post-intervention measurements immediately following the intervention, and a follow-up after 12 months. There are three study arms: control group (no advice), personalized sleep intervention (SL), and personalized nutritional intervention (NU). During the run-in period the following will be measured: sleep, physical activity, food intake, and glucose levels by continuous glucose monitoring (CGM) during 14 days covering night shifts, rest days, and non-night shifts. Information from this baseline will be used for the subsequent personalized advice. The intervention entails following the personalized advice for a period of three months.

The groups will be open-labeled. The intervention involves observable behavioral changes, of which the participants will be aware themselves. This makes it difficult to conceal whether a participant is receiving the intervention or not. Participants will have the opportunity to select their preferred intervention. This approach was chosen to optimize motivation for participants to adhere to the personalized intervention.

**Fig. 1:**
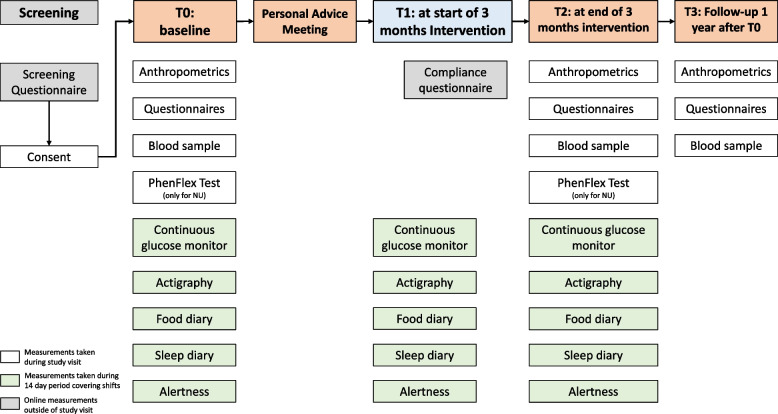
Schematic overview of the study. White represents measurements that will be gathered during a study visit. Green indicates measurements that will be taken during the 14-day period (covering night shifts, rest days, and non-night shifts) and monitored remotely. Grey indicates online measurements taken outside of a study visit. The follow-up occurs 12 months after the baseline visit. NU = nutritional intervention

The study has been approved by the Medical Ethical Review Committee of the Erasmus University Medical Center (Erasmus MC) and registered (27 November 2023) under ClinicalTrials.gov identifier NCT06147089. Participant recruitment is ongoing at the time of submission. An overview of the study setup is depicted in Fig. 1.

### Study population

The study population will consist of male employees who work at least 4 night shifts a month on average, with at least 1 year prior experience with shift work. Participants work in shifts with a duration of 6 h-12 h. A shift is categorized as a night shift if at least 1 h of working falls between midnight (00:00) and 06:00 in the morning. Participants can work night shifts only or work on rotating shifts. Participants are 18 to 60 years of age and without a diagnosis of a major disease. All participants live or work in the Netherlands and speak Dutch natively or fluently. The aim is to include 25 participants in each arm of the study.

### Sample size calculations

Sample size calculations were employed in GLIMMPSE 3.0, a validated manualized sample size calculator for longitudinal designs [33, 34]. We performed a repeated measure analysis sample size calculation using the Hotteling-Lawley Trace statistic to test for a time-by-treatment interaction effect, aiming for a power of 0.80 and a Type I error rate of 0.05.

For sleep outcomes, we predict a standard deviation of 84.4 min for total sleep time (TST), based on a laboratory study [35] comparing a control group with a sleep intervention. These estimates are based on large effect sizes, and it is unclear how these scores translate to a field study, therefore a mean scale factor was included to account for alternative values. Furthermore, we assumed that the Pearson correlation between all time-points was relatively stable at 0.48 based on a study on TST with a 2-year interval [36] and preliminary data from an Erasmus MC study on sleep and corona vaccination in shift workers (unpublished data, for protocol, see S-CORE study [37]). Taken together, for a desired power of 0.80 and a Type I error rate of 0.05, we estimated that we would need 18 participants per group for a difference of 128 min in TST as the main outcome in the sleep intervention arm.

Due to the paucity of previous data on continuous glucose levels of shift workers, there is no clear notion of the expected variability in a representative population sample. However, there is one observational study [38] that compared 62 daytime workers to 62 night shift workers who wore a continuous glucose monitor for 3–7 days, which showed standard deviation of 0.49 mmol/L for a mean nighttime glucose of 5.32 [38]. We assumed Pearson correlations of 0.85 between T0 and T1 and 0.71 between T1 and T2, considering patterns observed in Type I diabetes patients [39]. With these assumptions, we calculated a required sample size of 25 participants per group for a difference of 0.23 mmol/L in continuous glucose measurements as main outcome in the nutritional intervention. In total, considering both CGM and TST, our required sample size is *N* = 25 per group.

### Personalized intervention

**Fig. 2:**
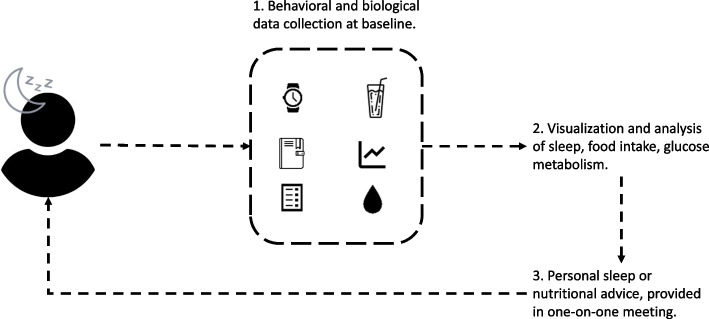
Baseline measurements are used to personalize the intervention. (1) Measurements collected during baseline, including sleep (via actigraphy watch, sleep diary, and sleep questionnaires), dietary behaviors (via food logging), and glucose metabolism (via continuous glucose monitoring and the Phenflex test with different blood based biomarkers measured pre- and postprandially). (2) These data and their interactions are visualized and analyzed to (3) personalize the sleep or nutritional advice

At the personal advice meeting (Fig. 1), participants will receive verbal and written instructions on their sleep or nutritional advice. In addition to providing the participant with information, motivational interviewing technique will be applied to increase adherence to the advice. It is a one-on-one meeting, where participants have ample time to ask questions. The personalization will be based on the individual circumstances of the participant (work schedule, commuting time, familial or social obligations), as well as (bio)markers of sleep and metabolic health (Fig. 2). The personal advice meeting will not include blood samples, questionnaires or anthropometrics, in order to lessen the study burden for the participants.

### Sleep intervention

The intervention will use multiple sleep episodes (split-sleep or naps) [11, 12], limiting prior wakefulness [13], and general guidelines on how to improve sleep for shift workers [40, 41], i.e. education on sleep hygiene, sleep and circadian pressure, planning of sleep, transitioning to day shifts/days off.

Baseline data from the run-in period will be used to generate personalized recommendations, including information on sleep quality and quantity (as measured by actigraphy and subjective questionnaire) and validated sleep questionnaires, like the Insomnia Severity Index (ISI) [42], Epworth Sleepiness Scale (ESS) [43], Pittsburgh Sleep Quality Index (PSQI) [44] and Shift Work Disorder questionnaire (SWD) [45]. These questionnaires can indicate a high vs. low-risk profile and differentiate for example between those who have trouble staying asleep vs. falling asleep. Lastly, social circumstances, as well as the opportunity to schedule extra sleep episodes within one’s schedule will be taken into account.

For instance, the tailored intervention can include using power naps during the night shift for those who have trouble staying awake during their night shift. Additionally, shift workers who wake up frequently, or have trouble falling back asleep, will be advised to wake up close to the start of their night shift to limit prior wakefulness.

### Nutritional intervention

The nutritional advice around nightshifts is built up in two steps. Firstly, advice on calorie distribution over 24 h will be provided. Specifically, participants will be advised to consume approximately 20% of their total daily caloric intake during a light midnight meal, 25% during breakfast (N.B. the end of the night shift but before sleep), 15% as an afternoon snack, and 40% during early dinner (early defined as 18:00–20:00 h) [23, 46]. Total daily caloric intake will be personalized based on individual estimated energy requirements [47].

Secondly, advice on macronutrient distribution over 24 h will be provided. Based on the fluctuations in insulin sensitivity throughout the day consumption of carbohydrates will be geared towards the morning, and fat intake towards the evening [23]. The advice entails that breakfast consists of 75% carbohydrates (CHO), 15% protein (PRO), and 10% fat (F), afternoon snack is 25% CHO, 25% PRO, 50% F, early dinner is 45% CHO, 20% PRO, 35% F, and light midnight meal is 55% CHO, 15% PRO, 30% F. Furthermore, overnight fasting and postprandial clinical biomarkers (i.e. the results of the PhenFlex Test and pre- and postprandial measurements of glucose, insulin, triglycerides, and cholesterol) will be used to personalize the advice related to intake of protein, carbohydrate and fat [48]. Advice on specific food products will also be provided, based on the outcomes of CGM and food logging. Lastly, personal dietary preferences will be taken into account (i.e. (dis)likes, halal, kosher).

### Outcome measurements

Data will be collected on anthropometrics, sleep, glycemic parameters, diet, biomarkers, and physical activity in night workers. Table 1 represents an overview of the outcome measurements.

**Table 1:** Research outcomes for the study protocol

Domain	Method of measurement	Parameters
Objective Sleep	Actigraphy	• Total Sleep Time (TST), Fragmentation index (FI), Wake after Sleep onset (WASO), mid-sleep • Sleep latency • Sleep efficiency
Subjective Sleep	Questionnaires	• Insomnia Severity Index (ISI) • Epworth Sleepiness Scale (ESS) • Pittsburgh Sleep Quality Index (PSQI) • Shift Work Disorder Questionnaire (SWD) • Circadian Type Inventory (CTI) • Chronotype
Blood Glucose Variability	Continuous glucose monitoring	• Time within ranges (hypoglycemic < 3.9 mmol/L, target 3.9–10 mmol/L or hyperglycemic > 10 mmol/L range) • Glycemic variability (coefficient of variation, CV) • Mean amplitude of glycemic excursions (MAGE) • Mean of daily differences (MODD)
Food intake	Food logging	• Diet quality measured by ‘Eetscore index’ Dutch healthy eating guidelines • Eating patterns: temporal distribution of energy intake from (macro)nutrients
Blood parameters	Concentrations of Biomarkers	• Glucose, insulin, HbA1c • Lipid profile: high-density lipoprotein (HDL), low-density lipoprotein (LDL), free fatty acids (FFA), triglycerides • Inflammatory markers: high sensitive C-reactive protein (hs-CRP), interferon-gamma (IFN-γ), Interleukin (IL)-6, adiponectin, leptin, Interferon gamma-induced protein 10 (IP-10) • Kidney Function: glomerular filtration rate (eGFR), creatinine • Liver function: aspartate aminotransferase (ASAT), alanine aminotransferase (ALAT), albumin, Gamma-glutamyl transferase (gGT) • Blood screen: hemoglobin (Hb), Hematocrit (Ht), mean corpuscular volume (MCV), leukocytes
Stress	Cortisol concentration and subjective questionnaire	• Hair cortisol concentrations • Perceived Stress Scale (PSS)
Alertness	Psychomotor Vigilance Test	• A 5-min validated psychomotor vigilance test recording reaction times (RT)
Phenflex *(only for NU)*	A mixed-meal challenge with blood samples (t0, t30, t60, t120, t240)	• Metabolic flexibility: dynamic response of glucose, insulin, free fatty acids (FFA), triglycerides, and cholesterol to the challenge • Inflammatory resilience: dynamic response of IL-6, IL-8, IL-10, tumor necrosis factor (TNF-α) to the challenge

### Primary endpoints

#### Quality and quantity of sleep

Participants will wear a lightweight combined actigraphy and accelerometer device (MotionWatch 8; CamNTech) on their non-dominant wrist for 14 days at T0, T1, and T2, to assess total sleep time (TST), Fragmentation Index (FI), wake after sleep onset (WASO), and mid-sleep. Actigraphy data will be supplemented with validated sleep questionnaires, measuring sleep quality (PSQI) [44], daytime sleepiness (ESS) [43], and insomnia (ISI) [42]. Follow-up measurements will only include the PSQI, ESS, and ISI (and not the actigraph). Additionally, participants will complete the Circadian Type Inventory (CTI) [49].

#### Continuous glucose patterns and food intake

Participants will wear a blinded CGM device (FreeStyle Libre Pro; Abbott) for 14 days at T0, T1, and T2. The CGM will measure glucose concentration in the interstitial fluid every minute, and readings are stored in 15-min intervals. CGM data will be used to calculate time within ranges (hypo-, hyperglycemic, or target ranges), glycemic variability, and intra- and inter-day differences. Participants will be asked to log their food intake for 3 days while working night shifts at T0, T1, and T2, using the mobile app “HowAmI” (TNO, the Netherlands, Version 1.1.34), in order to match food intake to CGM values. The HowAmI app uses the MyFatSecret database (Secret Industries Pty Ltd., Victoria, Australia) to record food intake and connects to a custom, parallel back-end database to record the time stamp for each meal. Additionally, participants will complete an index of diet quality, measured with the ‘Eetscore’ dietary assessment tool (developed by Wageningen University [50]) at T0, T2, and T3. The index is based on the Dutch Healthy Diet Index 2015.

### Secondary endpoints

#### Clinical biomarkers in blood

Blood levels of the following markers will be measured in all groups at T0, T2, and T3 from fasted blood samples: serum levels of glucose, insulin, HbA1c, lipid profile, kidney, liver function, blood screen, and inflammatory markers (for specifics, please see Table 1).

#### The PhenFlex Test

Participants in the NU group will drink the mixed-meal Phenflex Challenge Drink at T0 and T2. The Phenflex test is a beverage (440 ml) composed of lipids, carbohydrates, and protein corresponding to 950 kcal [51]. After an overnight fast, the beverage should be consumed in 5 to 15 min. Venous blood samples are collected via an intravenous cannula under fasting conditions (t = 0 min) and after ingestion of the PhenFlex drink (after 30, 60, 120, and 240 min). From the Phenflex test, it is possible to calculate metabolic and inflammatory resilience and to determine tissue-specific insulin resistance. Metabolic resilience is calculated from the dynamic measurements of glucose, insulin, free fatty acids (FFA), triglyceride (TG), and cholesterol, while inflammatory resilience is based on dynamic measurements of IL-6, -8, and -10, and Necrosis Factor Alpha (TNF-α).

### Anthropometrics

Anthropometric measurements will be done in all study groups at T0, T2, and T3 and include height (measured with Seca 213 Portable Height Measure), body weight (TANITA BC-730 or Seca 877 scale), and BMI. Hip- and waist circumference will be measured using measuring tape. Blood pressure will be measured using an automatic blood pressure monitor (e.g. Omron).

#### Objective and subjective alertness during night shifts

At T0, T1, and T2 participants will complete an alertness assessment involving a psychomotor vigilance task (PVT) on a mobile phone [52, 53], performed at the start and end of night shifts for 2 consecutive nights. The PVT is a visual reaction time (RT) test in which the participant is asked to maintain the fastest possible RTs to a simple visual stimulus [13, 54]. We will use a 5-min validated PVT which is suitable for the time-constrained environment of shift workers (NASA PVT + , USA, Version 1.2.0 or 1.4.4) [53] and training on the PVT during the baseline visit will minimize practice effects. Additionally, after completion of the PVT, sleepiness will be rated on the 9-point Karolinska Sleepiness Scale (KSS) [55].

#### Feeling of hunger, abdominal complaints, and faintness during night shifts

After the PVT during their night shifts, participants will complete a 6-item Visual Analog Scale (VAS) on hunger, fullness, desire to eat, thoughts of food, stomach upset, and feeling faint [23].

#### Stress markers

Hair cortisol reflects the cumulative secretion and long-term cortisol exposure. Therefore, we will cut a hair pluck of approximately 100 hairs from the posterior vertex of the scalp at T0, T2, and T3. Hair will be stored at room temperature in an envelope until analysis. Cortisol levels will be measured using an ISO 15189;2012-accredited LC–MS/MS method for hair cortisol analysis [56]. The method is described in detail elsewhere [57] and uses a Waters Xevo TQ-S system (Waters Corporation, Milford, MA, USA). Additionally, perceived stress will be assessed with the 4-item Perceived Stress Scale (PSS) [58] at T0, T2, and T3.

#### Quality of life

Quality of Life will be assessed with the WHOQOLBref questionnaire (26 items) at T0, T2, and T3, covering the dimensions life, health, energy, and personal satisfaction [59]. We will also examine work-life balance, via a 4-item validated questionnaire [60] at T0, T2, and T3.

### Covariates

#### Demographics

Information will be collected on age and sex at T0. Additionally, a comprehensive situation sketch will capture the social, work, and home environment at T0 (i.e. caregiver to a young child who sleeps/eats at certain times, very long commute to work, or very noisy neighborhood causing disrupted sleep). Lastly, participants will complete a one-item questionnaire [61] for chronotype (which is based upon the Munich ChronoType Questionnaire [62]) at T0.

#### Lifestyle habits

Self-reported information will be collected on smoking habits, average (weekly) alcohol intake, and medication use at T0 and T2.

#### Job characteristics

Participants will report on job type and work schedule (including deviations thereof) at T0, T1, and T2.

#### Subjective compliance with the intervention

Subjective compliance with the intervention will be assessed with a questionnaire in the SL and NU groups, during the intervention, after the intervention, and during the follow-up (to see if participants continued to use the advice after the intervention period ended).

### Statistical analysis

Our primary analysis will assess the changes over time (pre- and post-intervention) in sleep quantity, sleep quality and glucose regulation. The intra-individual differences within the NU and the SL group will be compared to the control group. By considering the intra-individual differences, participants can serve as their own controls, increasing the sensitivity of the analysis to potentially smaller changes.

Linear and logistic mixed models will be employed using statistical software (SPSS, R studio) to analyze the main effects over time for the factor condition (3 groups). A random intercept statement will account for inter-individual variations. Significance tests will be 2-tailed and at p ≤ 0.05. Covariates like age, sex, and BMI may be considered, and participants with baseline and at least one follow-up measurement will be included in the statistical analyses. Selective loss to follow-up will be analyzed through logistic regression. Covariates will be imputed using multiple imputation if needed.

## Discussion

The current study presents a controlled intervention involving personalized sleep and nutritional strategies for shift workers in a real-world setting. Previous studies have demonstrated a promising role for modifiable behavior in the prevention of disrupted sleep or glucose regulation in night shift workers [20, 23]. Despite these earlier studies, a notable gap still exists in our understanding of the effectiveness and long-term effect of these interventions in real-life settings.

The controlled design and comprehensive approach of this study will offer valuable insights into the multifaceted challenges encountered by shift workers. This encompasses the complexities of managing health and sleep amidst the demands of night shifts and individual and familial responsibilities. The findings of the GRIP study may serve as a foundation for refining and expanding interventions. For instance, if the findings indicate that adjusting the timing of food intake is both feasible and effective in mitigating negative health consequences, these findings could catalyze widespread adoption of interventions that target these modifiable behaviors. Furthermore, the identification of biomarkers linked to long-term circadian disturbance may be an important asset in monitoring the effects of such interventions.

In summary, this research deploys an array of biomarkers and wearable technology to examine the role of the circadian system in disease prevention for night shift workers, thus offering a unique contribution to the field of study. Understanding how personalized interventions impact the sleep and health of actual shift workers is not only essential for bridging knowledge gaps but also for crafting practical solutions that can potentially result in improved health, decreased healthcare expenses, and increased sustainable employability.

### Strengths & limitations

The first strength of this intervention is its diverse set of biomarkers, including markers of long-term health. In combination with the 1-year follow-up, this allows for an integrated view of altered physiological processes and contributes to the limited research on prevention of long-term health effects of shift work. Second, the intervention takes place in a real-life context. This enables us to explore the feasibility of the intervention within a day-to-day setting, as well as capture a high quality exposure assessment. With wearable devices such as the actigraphy watch and CGM, we can collect continuous objective data and combine this with actual information on worked shifts and food consumed. Especially the less-explored continuous processes are relevant, as they may provide a more concrete understanding of the underlying dynamics. Lastly, within the repeated measurements design participants can serve as their own controls, increasing the sensitivity of the analysis to potentially smaller changes.

Despite the strengths of this approach, there are potential pitfalls. First, participants will have the opportunity to select their preferred intervention. The researchers are aware that participants’ preferences might introduce a small confounding or selection bias to the data. However, the estimated risk for bias is judged to be low, as expected/putative confounding domains (i.e. chronotype, diet quality, activity level) will be measured validly and reliably and will be carefully controlled in the analysis. Moreover, the approach of individual preference might increase adherence and positive outcomes for participants. Indeed, allowing participants to select their preferred intervention, has been associated with beneficial outcomes, via an increased sense of autonomy within occupational health [63]. Furthermore, in the health promotion field, interventions that permit participants to select their preferred program consistently deliver the best outcomes in terms of health risk reduction, and are considered “best practice” [64]. Second, factors outside the scope of the study, such as workplace policies, personal lifestyle changes, or other external influences, might affect the results but could be challenging to account for. However, by inquiring about changes in the personal sphere or workplace at the follow-up meetings we expect to capture these external factors as well as possible, so that they may be estimated in our analysis. Third, during the course of the study, the changing of seasons could influence the circadian rhythm [65] of the participants due to variations in natural light exposure, temperature, and overall environmental conditions associated with different seasons. To mitigate this, careful consideration of seasonal changes (not only in the intervention groups but also in the control group) will be included in the results and their interpretation. Lastly, the inclusion of only male participants in our study hinders the generalizability to female nightshift workers.

## Conclusion

This study aims to improve sleep and metabolic health in night workers by introducing a personalized sleep or personalized nutritional intervention. The interventions are directed at alleviating both behavioral and physiological contributors to circadian rhythms and consequently minimizing health risks. Sleep, glucose regulation, biomarkers, and general health parameters will be evaluated. This study is unique in its personalized character, extensive combination of objective and subjective measurements and its long follow-up. This study will contribute to the current need for preventative health measures for night shift work in a real-life context.

## Data Availability

No datasets were generated or analysed during the current study.

## Abbreviations

EU: European Union
SL: Personalized sleep intervention
NU: Personalized nutritional intervention
CGM: Continuous glucose monitor
TST: Total sleep time
ISI: Insomnia severity index
ESS: Epworth sleepiness scale
PSQI: Pittsburgh sleep quality Index
SWD: Shift work disorder
CHO: Carbohydrates
PRO: Protein
F: Fat
FI: Fragmentation index
WASO: Wake after sleep onset
CTI: Circadian type inventory
FFA: Free fatty acids
TG: Triglyceride
TNF-α: Tumor necrosis factor alpha
PVT: Psychomotor vigilance test
RT: Reaction time
KSS: Karolinska sleepiness scale
VAS: Visual analogue scale
PSS: Perceived stress scale
CV: Coefficient of variation
MAGE: Mean amplitude of glycemic excursions
MODD: Mean of daily differences
HDL: High-density lipoprotein
LDL: Low-density lipoprotein
hs-CRP: High sensitive C-reactive protein
IFN-γ: Interferon gamma
IL: Interleukin
IP-10: Interferon gamma-induced protein 10
eGFR: Glomerular filtration rate
ASAT: Aspartate aminotransferase
ALAT: Alanine aminotransferase
gGT: Gamma-glutamyl transferase
Hb: Hemoglobin
Ht: Hematocrit
MCV: Mean corpuscular volume
